# Negative Selection Maintains Grossly Altered but Broadly Stable Karyotypes in Metastatic Colorectal Cancer

**DOI:** 10.1158/2159-8290.CD-24-0813

**Published:** 2026-01-21

**Authors:** William C.H. Cross, Salpie Nowinski, George D. Cresswell, Maximilian Mossner, Abhirup Banerjee, Bingxin Lu, Marc J. Williams, Georgios Vlachogiannis, Laura J. Gay, Ann-Marie Baker, Christopher Kimberley, Frederick J.H. Whiting, Hayley L. Belnoue-Davis, Pierre Martinez, Maria Traki, Viola Walther, Kane Smith, Javier Fernandez-Mateos, Erika Yara-Romero, Erica A. Oliveira, Salvatore Milite, Giulio Caravagna, Chela T. James, George Elia, Alison Berner, Chang-Ho Ryan Choi, Pradeep Ramagiri, Ritika Chauhan, Nik Matthews, Jamie Murphy, Anthony Antoniou, Susan K. Clark, Miriam Mitchison, Jo-Anne Chin Aleong, Enric Domingo, Inmaculada Spiteri, Stuart A.C. McDonald, Darryl Shibata, Miangela M. Laclé, Lai Mun Wang, Morgan Moorghen, Ian P.M. Tomlinson, Marco Novelli, Marnix Jansen, Alan Watson, Nicola Valeri, Nicholas A. Wright, John A. Bridgewater, Manuel Rodriguez-Justo, Chris P. Barnes, Hemant M. Kocher, Simon J. Leedham, Andrea Sottoriva, Trevor A. Graham

**Affiliations:** 1 Rare Diseases and Cancer Evolution Lab, Centre for Cancer Research, School of Biological Sciences, University of Reading, Reading, United Kingdom.; 2 Centre for Genomics and Computational Biology, Barts Cancer Institute, Barts and the London School of Medicine and Dentistry, Queen Mary University of London, London, United Kingdom.; 3 Centre for Evolution and Cancer, The Institute of Cancer Research, London, United Kingdom.; 4 St. Anna Children’s Cancer Research Institute (CCRI), Vienna, Austria.; 5 Department of Cell and Developmental Biology, University College London, London, United Kingdom.; 6 UCL Genetics Institute, University College London, London, United Kingdom.; 7 Computational Oncology, Department of Epidemiology and Biostatistics, Memorial Sloan Kettering Cancer Center, New York, New York.; 8 Department of Surgery and Cancer, Imperial College London, London, United Kingdom.; 9 Univ Lyon, Université Claude Bernard Lyon 1, INSERM 1052, CNRS 5286, Centre Léon Bérard, Cancer Research Center of Lyon, Lyon, France.; 10 Computational Biology Research Centre, Human Technopole, Milan, Italy.; 11 UCL Cancer Institute, UCL, London, United Kingdom.; 12 St. Mark’s Hospital, London, United Kingdom.; 13 St. George Hospital, Sydney, Australia.; 14 Tumour Profiling Unit (TPU), Institute of Cancer Research, London, United Kingdom.; 15 UCL Hospitals NHS Trust, London, United Kingdom.; 16 Barts Health NHS Trust, The Royal London Hospital, London, United Kingdom.; 17 Department of Oncology, University of Oxford, Oxford, United Kingdom.; 18 Department of Pathology, University of Southern California Keck School of Medicine, Los Angeles, California.; 19 Department of Pathology, University Medical Center Utrecht, Utrecht, the Netherlands.; 20 Wellcome Trust Centre Human Genetics, University of Oxford, Oxford, United Kingdom.; 21 Ludwig Institute, University of Oxford, Oxford, United Kingdom.; 22 Cancer Research UK Edinburgh Centre, MRC Institute of Genetics and Molecular Medicine, University of Edinburgh, Western General Hospital, Edinburgh, United Kingdom.; 23 Department of Gastroenterology, Barts Health, Whipps Cross Hospital, London, United Kingdom.; 24 Centre for Tumour Biology, Barts Cancer Institute, London, United Kingdom.

## Abstract

Aneuploidy is near-ubiquitous in cancer and contributes to tumor biology. However, the temporal evolutionary dynamics that select for aneuploidy remain uncharacterized. We performed longitudinal genomic analysis of 755 samples from 167 patients with colorectal-derived neoplasias from different stages through metastasis and treatment. Adenomas had few copy number alterations (CNA) and most were subclonal, whereas cancers had many clonal CNAs, suggesting that progression goes through a CNA bottleneck. Individual colorectal cancer glands from the same tumor had similar karyotypes, despite evidence of ongoing instability at the cell level. CNAs in metastatic lesions, after therapy, and in late recurrences were similar to the primary. Mathematical modeling indicated that these data are consistent with the action of negative selection on CNAs that “trap” cancer genomes on a fitness peak characterized by specific CNAs. Hence, progression to colorectal cancer requires traversing a rugged fitness landscape, whereas subsequent CNA evolution is constrained by negative selection.

**Significance:**

We profiled 167 long-term responders longitudinally (755 samples), documenting long-term cancer evolution. We found that a genetic bottleneck is required for progression and is associated with dramatic increase in CNAs but decrease in clonal diversity. After initiation, copy number evolution is constrained by negative selection through metastasis and treatment.

*See related commentary by Okada et al., p. 192*

## Teaser

Evolution by negative selection restrains genetic heterogeneity in colorectal cancer through metastasis and treatment, representing a new potential target for therapeutics.

## Introduction

Most cancer genomes are aneuploid (1). This is the result of gains and losses of whole or parts of chromosomes which are attained through a variety of mechanisms that include chromosome missegregation, aberrant double-strand break repair, and genome doubling (GD; 2). In colorectal cancer, approximately 85% of cancers are classified as chromosomally unstable (CIN) by virtue of having an abnormal karyotype and aneuploidy (3). The remaining ∼15% of cancers exhibit genetic instability at the mutational level and are classified as hypermutant but exhibit limited chromosome aberrations.

Aneuploidy likely has a causal role in cancer evolution. First, the observed ubiquity of aneuploidy in cancers and the prevalence of recurrent chromosome copy number alterations (CNA) are suggestive that some CNAs are positively selected (4, 5). Second, aneuploidy is predictive of progression to cancer in premalignant diseases such as Barrett’s esophagus (6) and ulcerative colitis (7). Third, in established cancers, individual CNAs have prognostic value over-and-above single-nucleotide alterations in key cancer driver genes (8). Fourth, ploidy has been shown to have prognostic value in many types of established cancer (9), and notably within-tumor heterogeneity of CNAs, as principally measured by the number of lineages with distinct karyotypes, is prognostic pan-cancer (10). In lung cancer, the diversity of CNAs but not single-nucleotide variants (SNV) has prognostic value (11); this has also been reported in prostate cancer (12).

Aneuploidy can also cause deleterious effects as the loss or gain of large genomic regions will affect the expression of thousands of genes and regulatory elements, some of which are likely to decrease tumor cell viability (13, 14). Moreover, it is likely that some chromosomal conformations prevent correct chromosomes segregation, resulting in cell death. Indeed, negative selection on CNA events has been previously reported (14, 15). At the same time, CNAs may be easy to acquire and with little adverse effects in cancer cells which are robust to genomic alterations. Chromosomal-scale alterations are therefore expected to result in trade-offs between the dosages of positively and negatively selected elements (16), potentially leading to an apparently stable aneuploid genome. Chromosomal changes may also have a buffering effect against deleterious point mutations (17). In such an evolutionary regimen, most alterations are either neutral or negatively selected (18). Indeed, studies show that the burden of CNAs is nonmonotonically related with prognosis across cancer types [first observed in breast cancer (10, 19)]. As such, cancers carrying an intermediate level of alterations (“just right” aneuploidy) are associated with worse prognosis than cancers with lower or higher levels of aneuploidy. Observing negative selection may be complicated by the fact that less fit variants are effectively removed from the population and do not come to clinical prominence.

The spatio-temporal evolutionary dynamics that produce the aneuploidy observed in cancer genomes remain poorly understood, in part because longitudinal measurement is often not clinically feasible and is compounded by the challenge of reconstructing ancestral history of chromosome gains and losses (20). Colorectal cancer presents a unique opportunity to track clonal evolution over space and time: precancerous lesions (colorectal adenomas) are occasionally “caught in the act” of transformation and are found to contain a small focus of cancer (early colorectal cancers). Colorectal cancer frequently metastasizes to the liver, and repeated hepatic metastatectomy can provide a source of longitudinally sampled tumor material amenable for molecular analysis.

## Results

### Benign-to-Malignant Transformation through a CNA Bottleneck

**Figure 1.**
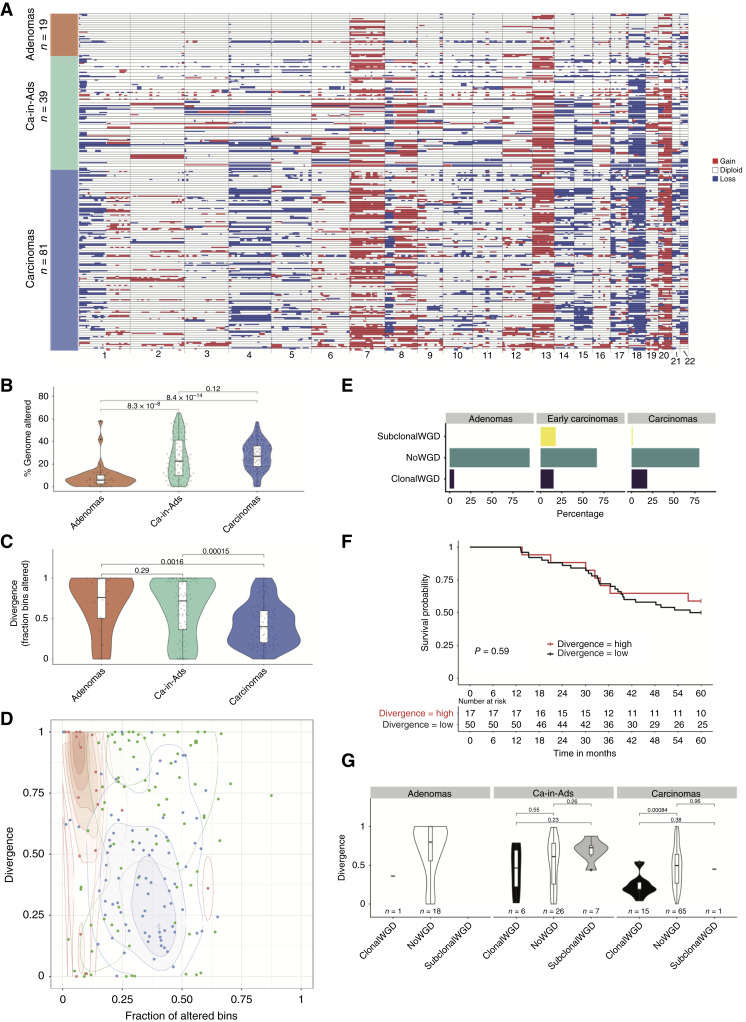
Aneuploidy increases through colorectal cancer progression, and clonal diversity decreases. **A,** Heatmap showing CNAs in adenomas (orange, top), early cancers (green, middle), and established cancers (blue, bottom). Thin black lines delineate cases, and within black lines are multiple regions of each case. GD status is shown on the left of the heatmap. Red = gain and blue = loss. **B,** PGA by adenomas, early cancers, and later-stage cancers. PGA increases through progression. **C,** The proportion of CNAs that are subclonal decreases through progression from adenomas to carcinomas. **D,** Phase space of PGA versus proportion of subclonal CNAs that are subclonal. Adenomas (orange) have low PGA but high CNA subclonality, whereas colorectal cancers (blue) have high PGA but low CNA subclonality. Early cancers (green) are more disparate in PGA and CNA subclonality. **E,** The proportion of CNAs that are subclonal is not significantly associated with overall survival in colorectal cancers (Kaplan–Meier analysis, groups split on the median proportion of subclonal CNAs across the cohort). **F,** Percentage of GD by tumor stage. GD is very rare in adenomas and more common in later stage cancers in which it is usually found to be clonal. **G,** The proportion of CNAs that are subclonal by tumor stage and GD status. Clonal GD is associated with a lower proportion of subclonal CNAs. ca-in-ad, cancer in adenoma.

In this study, we investigated the evolution of aneuploidy in a large cross-sectional and longitudinal cohort of adenomas and cancers. Previously, we observed that the degree of aneuploidy was much higher in a small cohort of (malignant) colorectal cancers than (benign) adenomas (21). To validate this observation, we performed multiregion spatial sampling and genome-wide CNA analysis [by shallow whole-genome sequencing (sWGS) or SNP-array] in *n* = 139 archival colorectal lesions (19 adenomas and 81 stage II/III colorectal cancers, termed “regular colorectal cancers”) and 39 early colorectal cancers of which 23/39 (59%) contained residual adenoma (Fig. 1A). In early colorectal cancers, adenoma and carcinoma regions were separately subsampled (minimum 2 regions per neoplasm). The percentage of genome altered (PGA) by a CNA was lowest in adenomas and highest in regular colorectal cancers (8.5% and 27.2%, respectively, *P* = 8.4 × 10^−14^) and early colorectal cancers (25.3%; Fig. 1B). Gains were more prevalent than losses in adenomas (*P* = 0.0052; Supplementary Fig. S1A), whereas they occurred at similar frequency relative to baseline ploidy in cancers (*P* = 0.75 and *P* = 0.32, respectively; Supplementary Fig. S1A). The CNA events that tended to be clonal or subclonal depended on tumor stage (Supplementary Fig. S1B).

CNA subclonal diversity (proportion of the total altered genome that is subclonal, i.e., alterated in at least one sample; “Methods”) was greatest in adenomas and low in regular colorectal cancers (*P* = 0.0016; Fig. 1C; Supplementary Tables S1 and S2). Different measures to quantify CNA subclonality were highly correlated (Supplementary Fig. S2). This suggests that progression from benign to malignant tumors goes through an aneuploidy-divergence “phase space” from low-aneuploidy/high-diversity to high-aneuploidy/low-diversity (Fig. 1D), indicating a selective genetic bottleneck acting on CNAs. Consistently, in early colorectal cancers, the fraction of subclonal CNAs was higher inside the adenoma part than inside the cancer part (*P* = 1.2 × 10−^6^; Supplementary Fig. S3A). Moreover, subclonal CNA fraction in the cancer part of early colorectal cancers was significantly lower than in late colorectal cancers (*P* = 0.046; Supplementary Fig. S3B). In short, as the tumor progresses intratumor heterogeneity decreases.

We trained a classifier to infer the presence of GD from sWGS data (“Methods”; Supplementary Fig. S4). GD was present in only a single adenoma and was clonal (Fig. 1E). In regular colorectal cancers, GD was more common (16/81, 18%) and was almost always clonal (15/16, 94%; Fig. 1E). Within early colorectal cancers, no adenoma component had GD, whereas a third of early cancers (13/39) had evidence of GD (*n* = 13/39; Fisher test *P* = 0.0011), and typically this was clonal (11/13, 85%). These observations suggest that GD occurs at, or shortly prior to, the time of malignancy. The proportion of subclonal CNAs was lower in GD than non-GD colorectal cancers (*P* = 0.00084; Fig. 1E), and divergent CNAs were significantly shorter in length (*P* = 0.015; Supplementary Fig. S5A). Therefore, following GD, the aneuploid genome accrues little further alteration within primary colorectal cancers, suggesting the process of GD produces a fit genotype which drives passage through the CNA bottleneck. We note this represents still an early event in the life of the carcinoma (indeed GD is largely clonal), right at the start of the cancer but likely toward the end of the premalignant phase.

We then investigated the relationship between CNA diversity and clinical outcome in regular colorectal cancers. The diversity of preexisting adaptive phenotypes in a population should correlate with population evolvability, and consequently more diverse populations are more likely to harbor an individual preadapted to a new selective pressure. Accordingly, intratumor clonal diversity has been observed as a pan-cancer prognostic biomarker (10), and it is prognostic in the premalignant lesion Barrett’s esophagus (22), lung cancer (11), and prostate cancer (12). However, in our cohort, the proportion of subclonal CNAs was not associated with overall survival (Fig. 1F; comparison of cases above and below the median proportion of subclonal CNAs ; *P* = 0.37; Fig. 1G; Supplementary Table S3). Consequently, these data suggested that intratumor CNA diversity did not strongly reflect the heterogeneity of adaptive phenotypes in regular colorectal cancer and was consistent with prior observations of a lack of positive selection at the subclonal level experienced by colorectal cancer (23, 24). The presence of GD was also not associated with overall survival (*P* = 0.56, Supplementary Fig. S5B).

### Limited CNA Diversity at Single-Gland Resolution in Colorectal Cancers

**Figure 2.**
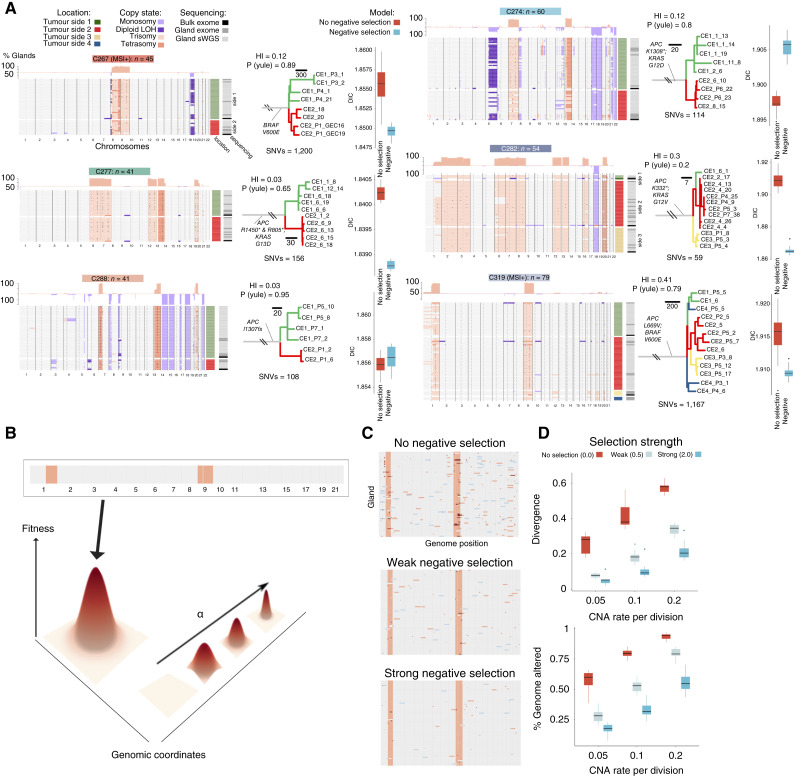
Lack of CNA heterogeneity at the level of individual colorectal cancer glands. **A,** (left column) Heatmaps showing CNAs in individual glands in each of six colorectal cancers. Glands were sequenced from arbitrary opposite sides of each colorectal cancer as indicated by the heatmap on the right side. Red hue colors indicate gains, and blue losses (center). Phylogenetic trees reconstructed from individual gland single-nucleotide alteration calls from exome sequenced glands. Trees are balanced (comparison with Yule model). Note, the trunk length is not shown to scale, and the number above the trunk represents the number of clonal SNVs. Branches are colored by notional tumor side where the gland was collected. HI = homoplasy index. Right, results of Bayesian inference to determine whether the observed CNA diversity is best supported by a model with or without negative selection. Model selection by deviance information criteria (DIC) suggests that the negative selection model is better supported for four patients (10 simulation replicates used for each boxplot). **B,** Schematic showing shape of fitness landscape used in the computational simulations of negative selection. The model parameter α determines the steepness of the single fitness peak in the landscape, at position defined by the above heatmap illustrating the optimal CNA pattern. **C,** Heatmaps showing CNAs of the randomly sampled glands in one simulation as function of selective strength α when the CNA rate is fixed (0.2). The simulations were based on data from patient C319, and 77 glands were sampled when the population size reached 100,000. **D,** Genetic divergence (top) and PGA (bottom) as a function of CNA rate and selection strength α (10 replicates for each case, the same simulation settings as in **C**). Strong selection on an initially optimal clone suppresses diversity, and CNA accumulation and higher mutation rates lead to more diversity and more CNA accumulation. The box plots show the median (center), first (bottom hinge), and third (top hinge) quartiles of the data; the whiskers extend to 1.5× of the IQR (distance between the first and third quartiles); data beyond the IQR are plotted individually.

Previous reports using FISH to measure CNAs at single-cell resolution found widespread chromosomal instability in colorectal cancer cell lines (25) and primary tumors (23). To investigate CNA heterogeneity at higher resolution we exploited the fact that well-to-moderately differentiated colorectal cancers are made up of a collection of glands, each composed of a few thousand cells recently derived from a common ancestor (23, 26). Glands are inevitably a small bulk sample but can reasonably be considered as units of selection in colorectal cancer, as gland fission likely expands the cancer cell population (23). We extracted a total of 307 individual colorectal cancer glands from the notional “left” and “right” opposite sides of an additional six regular colorectal cancers (Fig. 2A; Supplementary Table S4) and performed high-depth exome sequencing (59 glands) or sWGS (248 glands, few 1,000s of cells per gland). In addition, we also analyzed at least two bulk samples composed of numerous glands from each tumor.

At gland-level (individual clone) resolution, the pattern of CNAs was quite similar within cancers (Fig. 2A; Supplementary Tables S5 and S6). In all cases, a “core karyotype” was evident across the glands from that case, with a small numbers of glands showing additional subclonal CNAs (mean CNAs per gland = 7, mean clonal CNAs = 6.5). We note that intratumor CNA heterogeneity was detected, but we emphasize the clonality of most CNAs. Zooming in further, single-cell analysis by FISH in four additional cases (Supplementary Fig. S6A–S6D) and single-cell sequencing in case C274 [reported in Bollen and colleagues (27)] confirmed ongoing instability at the level of individual cancer cells within glands.

SNVs were called from individual gland exome sequencing data and used to reconstruct phylogenetic trees (“Methods”; Fig. 2A; Supplementary Table S7). Trees were balanced (ancestral nodes have equal numbers of offspring; Yule model; *P* > 0.05 for all cases), suggestive of a lack of stringent subclonal positive selection, in accordance with previous reports (23, 24). Individual glands from (Microsatellite Stable) MSS tumors contained on average 156 SNVs (range, 68–232) which equated to 40 more SNVs than the most recent common ancestor (MRCA) cell of the cancer (371 SNVs for hypermutant cases). We calculated the SNV accumulation rate per year using only C>T mutations in the four trinucleotide contexts associated with COSMIC mutational signature 1 (ACG, CCG, GCG, and TCG) which are known to accrue at a constant rate in colorectal cancer (28). This analysis produced an estimate of the time to the MRCA of 24 years (range, 11–30 years). Despite this elapsed time, we observed little subclonal accrual of CNAs within the cancers.

### Mathematical Modeling Negative Selection on CNAs

We hypothesized that the distinct pattern of CNAs observed in each colorectal cancer represented a (local) optimum in the fitness landscape, with negative (purifying) selection suppressing diversity at gland and bulk levels. To test this hypothesis, we developed a mathematical model of CNA accrual during cancer growth and the evolutionary dynamics due to the action of negative selection on the CNAs. Individuals in the model (“glands”) had variable fitness ($s=\frac{b_{i}\;\;-{\;d}_{i}}{b_{o}\;\;-{\;d}_{o}}-1$) defined by the gland’s karyotype according to the following relation:

$$b_{i}-d_{i}=\frac{b_{o}\;-{\;d}_{o}}{1\;+\;\alpha \;*\;d\left(G_{i},\;{\;G}_{o}\right)}$$

In which $b_{i}$ ($d_{i}$) is the birth (death) rate of gland $i,\;\;G_{i}$ is the karyotype of gland i, $G_{o}$ is a prespecified “optimal” karyotype that maximized fitness, $b_{o}$ ($d_{o}$) is the “optimal” birth (death) rate, and the function $d\left(G_{i},G_{o}\right)$ measured the genetic distance between the two karyotypes. A gland’s karyotype was represented by a vector of length C, in which C is the number of bins used in our empirical data analysis, with each entry representing the integer copy number of each locus in the gland’s (abstracted) genome. The number of CNAs followed Poisson distribution at mutation rate μ per gland division, in which the size of each CNA (in the unit of bins) was sampled from the sizes of observed CNAs in our data. A gland’s fitness was then inversely proportional to the absolute distance between a gland’s karyotype and the maximum fitness karyotype ($G_{o}$); the parameter α scaled the strength of negative selection experienced by nonoptimal karyotypes, with α=0 defining a “flat” landscape in which all karyotypes have the same fitness (i.e., no negative selection), $s=s_{\max}$ (Fig. 2B; Supplementary Modeling Note).

Stochastic simulations showed that ongoing mutation within a growing tumor in the absence of negative selection (α=0) generated high CNA heterogeneity (Fig. 2C and D). A growing tumor that initially occupied the fitness maxima (karyotype of the first cancer gland = $G_{o}$) experienced negative selection which suppressed CNA diversity, with stronger negative selection (greater α) more severely limiting diversity (Fig. 2C and D) and higher mutation rates generating more diversity (Fig. 2D). Thus, modeling suggested that negative selection for an optimal CNA karyotype was a plausible explanation of the apparent stability of the aneuploid genome observed in colorectal cancer glands. Performing simulations from a diploid starting point further demonstrated convergence on an optimal karyotype within a meaningful time frame (population size, *N* = 50,000, Supplementary Fig. S7A–S7E), even when experiencing a 10-cell bottleneck that may occur, for example, during metastasis.

Our previous single-cell sequencing data, derived from cells extracted from colorectal glands from the same tumors analyzed in this study (27), provided an empirical measurement of the lower limit of the CNA rate at μ≈0.1 new CNAs per gland division. This prior information was important as mutational rate and selection are nonidentifiable in a stochastic branching process model (Supplementary Fig. S8A–S8C). The dynamics of cells within glands affect the scaling of per-cell to per-gland mutation rates in a complex fashion dependent on the mechanism of selection upon cell/gland karyotypes; for simplicity we neglected to consider these dynamics and took prior distributions that set per-gland mutation rates the same as cellular rates. We used Bayesian model selection to compare the fit of models with negative selection versus no selection (purely neutral evolution) on the six cancers subjected to gland-by-gland analysis. In 4/6 cancers the model with negative selection better represented the data than the model without negative selection (Fig. 2A), and it was not possible to distinguish between the models in 1/6 cases. The model rests on a number of assumptions, and the goodness of these assumptions (see “Discussion”) determines the strength of the conclusion that can be drawn from these analyses. Nevertheless, modeling provided some quantitative evidence that the low levels of CNAs diversity in primary colorectal cancers was consistent with the acting of negative selection upon a core karyotype.

### Ongoing CNA Accrual in Colorectal Cancer Model Systems

Our model of negative selection presumes that CNA events were constantly generated during tumor development and were purged from the population by negative selection, leading to minimal CNA heterogeneity. An alternative hypothesis is that CNA alteration rates are very low most of the time, possibly with occasional bursts of high CNA rates that produced the observed highly altered genomes. We note that this is not necessarily the same as *punctuated evolution*, which does not require bursts of mutation rates because phenotypes develop in peripherally isolated populations in separated environmental niches (29). High ongoing CNA rates in cancer and specifically in colorectal malignancies, rather than a burst of CNA events, has been demonstrated previously (27) and was supported by single-cell assays in our dataset (Supplementary Fig. S6A–S6D; ref. 27).

**Figure 3.**
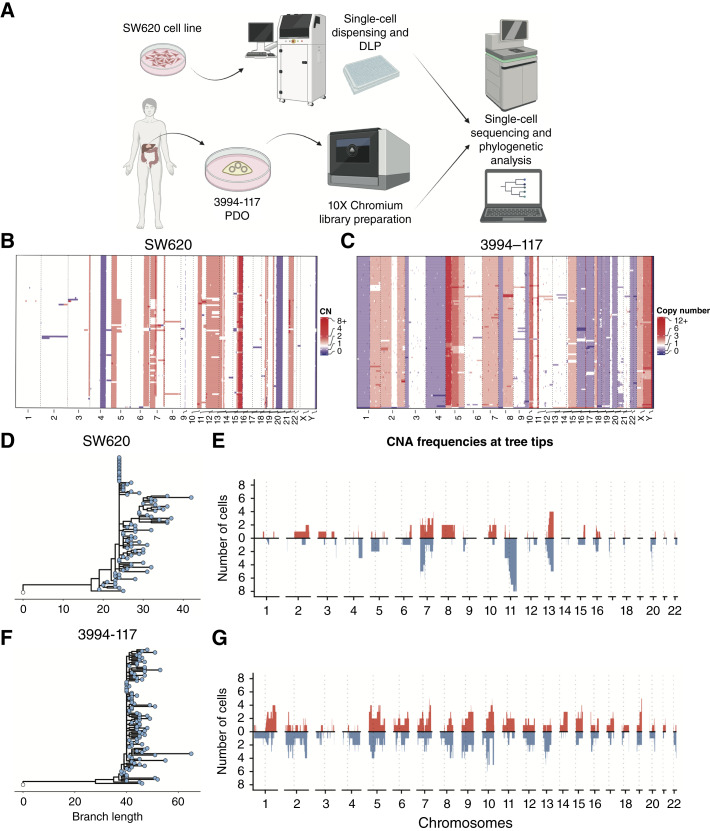
Ongoing copy number alterations in model systems. **A,** Schematic of the method used to obtain and sequence single cells from cultured colorectal cancer cell line SW620 and patient-derived organoid (PDO) 3994-1117. Cell line SW620 underwent single-cell dispensing using the cellenONE system, followed by direct library prep (DLP) while the 10× Genomics Chromium was used to prepare single-cell libraries from the patient-derived organoid 3994-1117. **B,** Heatmap showing CNAs in single cells from colorectal cancer cell line SW620 and (**C**) from patient-derived organoid 3994-1117. Red = gain, blue = loss. **D,** Phylogenetic tree constructed using CNAs in the colorectal cancer cell line SW620. **E,** Frequency of copy number gains and losses at the tips of the SW620 phylogenetic tree. **F,** Phylogenetic tree constructed using CNAs in the colorectal cancer organoid 3994-117. **G,** Frequency of copy number gains and losses at the tips of the 3994-117 phylogenetic tree (as in **E**).

We sought to provide further evidence of an ongoing high CNA rate in colorectal cancer. We generated single-cell DNA sequencing (scDNA-seq) data from SW620 colon cancer cells (Fig. 3A; Supplementary Table S8). Using 91 high-quality cells (see “Methods”), we performed CNA calling and phylogenetic analyses. This highlighted that individual cells had ongoing chromosomal instability because the cells had generated multiple new CNAs since their MRCA, in addition to a number of shared (clonal) CNAs in the population (Fig. 3B). The new CNAs were spread across the genome, and phylogenetic analysis showed that cell-unique (tip) CNAs were enriched for chromosome 11 losses. This may indicate that the copy number gain state (copy number = 3) in chromosome 11 is unstable and frequently reversed in SW620 cells or could indicate selection for this loss in our cell culture conditions.

We next performed scDNA-seq in a patient-derived colorectal cancer organoid (30), which also showed ongoing CNA generation in individual cells that were spread across the entire genome (Fig. 3C; Supplementary Table S8). Phylogenetic analysis of CNAs with MEDICC2 (31) showed ongoing CNAs in both models (Fig. 3D–G), although in the organoid model, the tree structure was very balanced and there was a lack of recurrent copy number events that was particularly consistent with lack of positive subclonal selection (Fig. 3F and G). Hence, CIN is ongoing at the single-cell level but does not manifest macroscopically because of negative selection that does not let clones survive and expand.

### Negative Selection on CNAs Continues through Metastatic Dissemination and Treatment

We then investigated how CNAs evolved during metastatic spread and treatment. Colonization of a new tissue and the application of therapy represent new selective pressures that could change the core karyotype observed in primary colorectal cancers. Even in the absence of positive selection, bottlenecks during metastatic colonization combined with the strong effects of genetic drift in an initial small population, could lead to gross differences between primary tumor and metastatic deposits (i.e., founder effect). Colorectal cancer frequently metastasizes to the liver and less frequently to other organs, including the lung. We collected tissue from 23 primary sites and 68 metastatic lesions from 22 patients, with the material collected longitudinally from a mode of two time points (range, 1–5 time points). Five patients remained untreated throughout their studied time course (an additional three patients received no treatment between sampled time points), whereas other patients had experienced a diverse range of treatment regimens (Supplementary Table S9). Using sWGS, we measured inferred absolute copy number of CNAs for each tumor deposit (total of 168 distinct regions analyzed, Supplementary Table S10) and called GD as described above. Comparing metastatic lesions with our previous primary colorectal cancer cohort (Fig. 1), we observed that CNA diversity only fractionally increased in the metastatic cohort (Supplementary Fig. S9A). The frequency of GD was significantly higher in metastatic cases [18/22 (82%) had GD] versus regular colorectal cancers [16/81 (20%) cases GD; *P* < 0.0001; Supplementary Fig. S9B] and was comparable with previous reports of GD in metastatic colorectal cancer (32). GD was clonal in two thirds of metastatic cases with GD (12/18; 66%).

**Figure 4.**
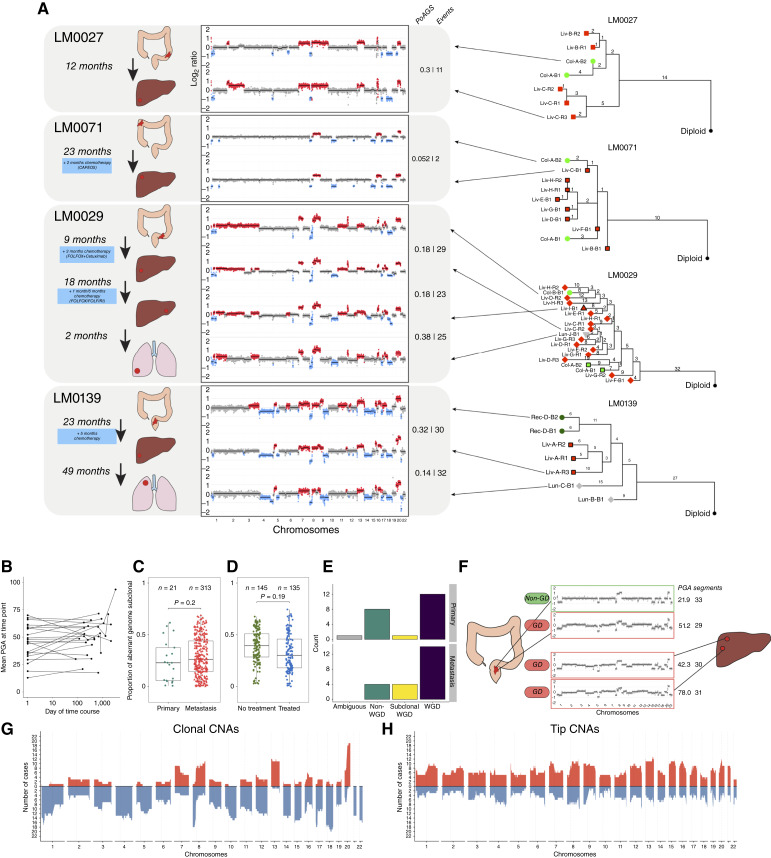
Stability of CNAs across space, time, tissue and treatment in colorectal cancer. **A,** Longitudinal assessment of CNAs in primary colorectal cancers and metachronous metastatic lesions in liver and lung showed similar CNA profiles were observed over space time, tissue and through various treatment courses. Timelines of four representative metastatic patients are shown. Schematics show the organ where cancer deposit was sampled. Genome-wide view of CNAs with gains (red) and losses (blue) relative to baseline ploidy. Proportion of Aberrant Genome Subclonal (PoAGS) and number of events are displayed for each comparison presented sequentially. To the right of each timeline is the corresponding phylogenetic tree as described in Supplementary Fig. S10. **B,** Mean PGA averaged across all lesions at the sampling time by time since baseline (removal of the primary colorectal cancer). The PGA remained broadly constant over time in each patient (*x*-axis is days and is on a log scale). **C,** The pairwise difference in CNAs between regions of the primary tumors was not significantly different to the pairwise difference between regions of paired metastatic lesions. **D,** Across time points genetic divergence was not significantly different after treatment with various chemotherapy and/or targeted agents. **E,** GD was common in metastatic primary tumors and metastatic deposits and was sometimes present in some but not all of the metastatic lesions from a patient (called subclonal GD). **F,** An example case (LM0032) where GD was subclonal in the primary but clonal in the metastasis. The greater burden of CNAs is evident in the GD samples. **G,** Frequency of copy number gains (red) and losses (blue) present on the clonal branches in the set of phylogenetic trees created from the cohort. Over-representation of some CNAs is evident. **H,** Frequency of copy number gains and losses at the tips of the phylogenetic trees (i.e., unique events). CNAs are uniformly spread across the genome.

We examined how CNAs evolved over space and time by comparing patterns of CNAs within and between metastatic lesions and with the primary tumor. Additionally, we performed phylogenetic analysis per patient in the cohort based on CNAs using MEDICC2 (Supplementary Fig. S10). We observed several examples of the maintenance of a core abnormal karyotype in the primary tumor wherein the pattern of CNAs did not greatly change in metastatic lesions through space, time, and metastasis to other tissues and through chemotherapy and/or targeted treatment (Fig. 4A–D; Supplementary Figs. S11–S13). In nearly all cases, there were some CNA differences between lesions in the same patient, and we do not discount that these differences may be important for tumor cell biology.

We measured the proportion of the aberrant genome that was subclonal in pairwise sample comparisons within each patient (pairwise to remove the effect of extensively sampled tumors increasing subclonality), the mean of which we term divergence, in addition to counting phylogenetic events (we note the two are highly correlated, Supplementary Fig. S14). Patient LM0027 displayed little divergence in karyotype between the primary tumor and a metastasis taken a year later in the liver, with no treatment in between [Fig. 4A; divergence of 0.26 (0.18–0.31), mean absolute PGA difference 5.4% points (pp), and mean number of phylogenetic events 11 (4–17)]. In patient LM0071, despite 23 months between the primary colon and metastatic liver samples and 3 months of oxaliplatin and capecitabine chemotherapy, maintenance of a core karyotype was observed [Fig. 4A; divergence 0.17 (0.03–0.22), mean absolute PGA difference 1.6 pp, and mean number of events 5.1 (2–7)]. LM0029 was followed for 28 months from primary diagnosis, through hepatic metastatectomies, one pulmonary metastatectomy, and three challenges of chemotherapy in combination with anti-EGF mAb cetuximab. Again, the core karyotype was broadly maintained throughout, with the largest difference seen in the lung metastases which lacked a gain of chromosome 2 [Fig. 4A, divergence between consecutive sampling points of 0.19 (0.13–0.25), 0.34 (0.19–0.60), 0.26 (0.12–0.52), and 0.38, respectively; mean absolute PGA differences 4.6, 6.1, 6.2, and 18 pp, respectively; and mean number of events, 33 (30–36), 27 (14–38), 25 (13–40), and 25 (25–25), respectively]. Similarly, in patient LM0139, resection of the primary colorectal cancer at baseline was followed by hepatic metastatectomy at 23 months and pulmonary metastatectomy at 72 months. Despite 6 years of elapsed evolution and metastasis to two separate organs, the final karyotype sampled in the lung had a very similar pattern of CNAs to the initial karyotype observed in the primary, demonstrating marked stability across time and space [divergence between the first and last time points 0.28 (0.18–0.38), mean absolute PGA difference 12 pp, and mean number of events 36 (35–36); Supplementary Fig. S15A].

Despite these examples of relative stability, we observed some cases in which CNA patterns diverged markedly between lesions. For example, the metastatic lesions in the ovary and omentum sampled at the second time point in LM0018, taken only 7 months after the sampling of the primary, showed the highest between time point divergence in the cohort [divergence 0.62 (0.55–0.7), mean absolute PGA difference 27 pp, and mean number of events 42 (32–52), Supplementary Fig. S11]. The patient received FOLFOX in between sampling points. These samples were our only examples of ovary and omentum spread but raise the possibility that CNAs may change substantially to enable colonization of these organs. We also highlight case LM0011 which also showed a large increase in divergence between the two time points in the colon and liver taken 16 months apart despite receiving no treatment in the interval [divergence 0.53 (0.49–0.62), mean absolute PGA difference 21 pp, and mean number of events 52 (40–67), Supplementary Fig. S11].

Alterations in the PGA were generally low in sequential time points, indicating aneuploidy stability (mean PGA increased by 3.4 pp on average for consecutive time points in the cohort; Fig. 4B). Within the same time point, CNA differences between metastatic regions was not significantly different from CNA differences between primary regions (Fig. 4C; *P* = 0.2, linear mixed model, Supplementary Fig. S15B), and divergence within regions of primary colorectal samples was consistent with our analysis of regular colorectal cancers (divergence 0.24). Divergence between primary sites and the liver was greater than same-site comparisons (Supplementary Fig. S15C); additionally, divergence between lesions at the same time point was significantly less than across time points (Supplementary Fig. S15D). Overall, CNA divergence was not significantly different in on-treatment intervals versus off-treatment intervals (*P* = 0.19, linear mixed model, Fig. 4D). Additionally, mean PGA change in treated and untreated intervals was similar (Supplementary Fig. S15E). Considering the time elapsed during intervals showed that divergence remained unaffected by treatment on a cohort level (Supplementary Fig. S15F and S15G), although the change of PGA per month was significantly greater than 0 in treated intervals (Supplementary Fig. S15H).

Subclonal GD was inferred in six patients (LM0018, LM0019, LM0029, LM0032, LM0056, and LM0124), and in these cases, GD was enriched in metastatic samples versus primaries (Fig. 4E). For example, the metastatic samples in LM0032 were GD, whereas one of the two primary samples was not, suggesting that GD was subclonal at the primary site and potentially led to the metastatic clone (Fig. 4F). In LM0056 and LM0124, all primary samples were classified as non-GD, yet all bar one metastasis sample in each patient was considered GD, indicating that the GD likely originated after metastasis. Only a single liver metastasis region was called as GD in LM0019. In primary colorectal cancers, GD tumors had less diverse CNAs. The reason for this difference could be related to elapsed time: in GD, primaries could be fit and grow rapidly with little elapsed time for new CNA evolution despite an intrinsically higher mutation rate, whereas in metastasis, sufficient time elapses for new CNAs to accrue. Divergence also increased with ploidy inferred during copy number calling in the metastasis cohort (Supplementary Fig. S15I).

Based on the phylogenetic structure inferred for all 22 patients, we separated CNA events into three categories: clonal (events preceding the MRCA of the tumor samples), intermediate (events shared between samples but not preceding the MRCA), and tip (events unique to each sample). Clonal CNAs had occurred early in the cancer’s development, and so selection had had time to operate on these events. In comparison, tip CNAs had occurred more recently and so may not yet have been assorted by selection. The distribution of clonal events across the genome showed that some CNAs were far more common than others, consistent with the typical pattern for colorectal cancer (33). Events such as 8q, 13 and 20 gain, as well as 8p and 17p loss were enriched only in clonal alterations (Fig. 4G). Contrastingly, the events at the tips of the phylogenetic trees (Fig. 4H) showed a strikingly uniform distribution across the genome, suggesting an ongoing accrual of CNAs at a constant rate across the genome prior to assortment by selection. We also explicitly compared the distribution of CNAs in primary versus metastatic liver samples and confirmed that no significant enrichment for metastasis-specific CNAs was found, again supporting the finding that CNA profiles are stable also across the metastatic process (Supplementary Fig. S16A and S16B). Comparing early colorectal cancers (Supplementary Fig. S17A) versus primaries from the metastatic cohort stages I to III at diagnosis (Supplementary Fig. S17B) also did not reveal differences in recurrent events, although PGA (but not the number of copy number segments) was higher for primaries that metastasized (Supplementary Fig. S17C and S17D).

## Discussion

Our study reveals the dynamics of CNAs during the evolution of colorectal cancer. We observe large changes in CNAs during the progression from benign to malignant disease, but thereafter the cancer karyotype seems quite stable and typically remains relatively unchanged over many years, through metastasis to distant organs and despite treatment. Taken together, these data suggested a model whereby strong positive selection for an abnormal karyotype occurs during transformation from benign to malignant disease and that thereafter the karyotype evolution is constrained by negative selection acting at the level of individual cells, including in metastasis and during treatment. This is a distinct mechanism to transient bursts in the CNA rate as has been previously suggested to occur in breast cancer evolution (34). We note that our finding of pervasive negative selection at CNA level is congruent with our previous observation of “Big Bang” effectively neutral evolution in colorectal cancers (23, 24), because negative selection acts to remove deleterious variants before they can expand to detectable levels and so only neutrally evolving lineages remain. Positively selected variants, both SNVs and CNAs, are clonal in the tumor and part of the “trunk” of the phylogenetic tree but do not drive subclone expansions.

We propose that an altered “core karyotype” is necessary for the initiation of malignancy and that the same karyotype may be sufficient for subsequent metastatic spread in colorectal malignancies. This differs in other cancer types, in which metastatic spread has been previously linked to specific aneuploidies (11, 35). In colorectal cancer, these evolutionary dynamics are consistent with the observation of early metastasis (36), whereby the genome of the initial primary tumor is competent for metastasis. Given the intertumor heterogeneity in patterns of CNAs, we suggest that the fitness landscape traversed by CNAs is “rugged,” with local fitness peaks located at normal diploid cells and at multiple aberrant-genome positions, and that fitness of any individual karyotype is likely to be somewhat patient-specific due to the background of germline and somatic variants and tumor microenvironment composition. There could be multiple local maxima close together in genotype space—or similarly new CNAs could be deleterious or neutral—our analysis is insensitive to detect these dynamics. Our data do suggest that fitness peaks are likely sufficiently distant in karyotype space to prevent easy transition between peaks and potentially that the selective landscape of CNAs differs between benign and malignant disease. Hence, whereas during carcinogenesis there is certainly positive selection for some CNAs, there is also strong *negative selection* of alterations that deviate from a “core karyotype.” This is not the same as conserving the accumulated drivers. Indeed, accumulating functional evidence suggests that, apart from focal high-level amplifications (which are rare in colorectal cancer) and loss of heterozygosity events for tumor-suppressor genes, the number of CNA events that are under positive selection is relatively low, as it is for driver point mutations [e.g., Girish and colleagues (37)]. For instance, a likely cause of constrains in the stability of a karyotype could also be the necessity of being able to properly segregate chromosomes or maintaining a functional chromatin state in the 3D genome.

Given the potentially defining role of CNAs in colorectal cancer evolution, we hypothesize that the fixed pattern of CNAs in any individual colorectal cancer likely defines the biology of that tumor and consequently is a major determinant of patient prognosis and treatment response. That the karyotype is relatively fixed through treatment is surprising, as presumably the effectiveness of therapy is dependent on it acting as a novel and strong selective pressure. We had expected this new selective pressure to change the fitness landscape and as a result cause wholesale change in the CNAs observed after therapy. It may be that the fitness peak of maximal treatment resistance is too far from that occupied by an untreated tumor, and so is highly unlikely to evolve. However, we emphasize that our analysis has taken a genome-wide average view, and adaption could be driven by individual and/or small CNAs (8, 38) for which our analysis is insensitive to detect. Nevertheless, an alternative explanation is that chemotherapy response in colorectal cancer may be entirely plastic (39) and so does not involve the clonal selection of individual lineages–a phenomenon that may be common across cancer types (40). In colorectal cancer, previous work is suggestive that plasticity is established very early in colorectal cancer (40) and is consistent with reports of early metastatic spread (36).

We reiterate that there are typically clear genetic differences between matched primaries and metastases, and we do not rule out that these differences could contribute importantly to tumor biology: we recognize that our analysis is insensitive to detect a driving role for such events. We acknowledge that our study is limited by its principal reliance upon bulk sampling, including our use of individual tumor glands, and stronger evidence for or against negative selection on CNAs will derive from large-scale single-cell sequencing analyses. Our modeling relies on a number of critical but untested assumptions, such as simple scaling of mutation rates between tumor cells and glands, and steady division rates of disseminated tumor cells. All these assumptions can alter the expectation of the degree of genetic divergence between and within lesions. Appropriately addressing these assumptions is a major undertaking and should be a priority for future work. Moreover, in this study, we focus on colorectal cancer, but it is evident that levels of CIN and intratumor heterogeneity and their correlation with survival or importance in cancer biology is likely to vary significantly between cancer types, and our statements may not generalize beyond colorectal cancer.

Collectively, our study demonstrates a plausible role for negative selection acting on the karyotype to define the genomes of colorectal cancers.

## Methods

### Patient Sample Acquisition and Processing

1. Archival samples.
2. Various cohorts of archival specimens were used in this study:
  a) Oxford: Colorectal adenomas, colorectal cancers, and cancer in adenoma (ca-in-ad) specimens used in Fig. 1 were obtained from the pathology archives of the John Radcliffe Hospital under ethical approval from Oxfordshire REC A (MREC 10/H0604/72).
  b) Utrecht: 26 early cancer lesions used in Fig. 1 were obtained from the University Medical Center in Utrecht. These samples were extracted from an earlier cohort-nested matched case–control study on pedunculated T1 colorectal cancers conducted in the T1 colorectal cancer registration cohort, initiated by the Dutch T1 Colorectal Cancer Working Group, a collaboration of Dutch hospitals, including patients with T1 colorectal cancer diagnosed between January 1, 2000 and December 31, 2014, identified using the Netherlands Cancer Registry. The earlier study was approved by the Medical Ethics Review Committee of the University Medical Center Utrecht (approval for data collection and histologic review, reference number: 15–487 and 15–716) and performed in accordance with the Helsinki Declaration.
  c) Barts: Metastatic colorectal cancer samples used in Fig. 4 were obtained from the Barts Cancer Tissue Bank (www.cancertissuebank.org; REC Ref: 14/LO/2031; tissue request reference number 2015/2/QM/TG/CaCOL). After screening the records of 200 patients in the Comparative Toxicogenomics Database (CTB) database, 22 patients were chosen who had all undergone surgical resection of the primary colorectal cancer and also had liver metastases resected. Subsequently, some of these patients also underwent resection of extrahepatic metastases (e.g., lung) or recurrent liver metastases. All relevant clinical information associated with these patients was collected by performing a thorough search of the NHS electronic patient records and the CTB database.
3. Fresh-Frozen Samples

Fresh-frozen colorectal cancer samples used in Fig. 2 were collected from University College London Hospitals under ethical approval 11/LO/1613 and via the University College London Hospitals Biobank (15/YH/0311). All samples were collected from patients who had given informed consent. Cancers were obtained from surgical resections and processed within the same day. A maximum of four different spatial regions (biopsies) were sampled from each tumor, and these were further separated into smaller tissue pieces and subsequently cryopreserved (medium: minimum essential medium, 10% DMSO, 5% FCS, and 25 μmol/L HEPES buffer). Individual glands were pulled from thawed tissue pieces using a stereoscopic microscope (Zeiss Stemi SVII microscope) and transferred and stored in buffer ATL (Qiagen) until DNA extraction.

1. Patient-Derived Organoid Samples

Written informed consent was obtained from patients. Studies were conducted in accordance with Declaration of Helsinki. Studies were approved by an Institutional Review Board (Prospect C: National Research Ethics Service: 12/LO/0914; Prospect R: Medicines and Healthcare products Regulatory Agency: 15983/0249/001–0001).

### Tissue Processing, DNA Extraction, and Quality Control

1. Archival Samples

For cases used in Fig. 1: Formalin-fixed, paraffin-embedded (FFPE) blocks were sectioned onto glass slides at 10 μm thickness, and following dewaxing, regions of interest were scraped into tissue lysis buffer using a pathologist-annotated hematoxylin and eosin slide as a guide.

For cases used in Fig. 4: FFPE blocks were sectioned at 8 μm thickness onto PALM (Zeiss) slides. A serial hematoxylin and eosin slide was reviewed by one of the collaborating pathologists to confirm tumor content and guide subsequent isolation of cancer tissue. A combination of techniques was used to obtain tumor tissue from the slides: when tumor cell content was high material was scraped into tissue lysis buffer (most primary colorectal cancers had high tumor cell content). When tumor cellularity was intermediate, the PALM MicroBeam laser capture microdissection system (Zeiss) was used to isolate regions enriched for tumor cells, and these regions were gently lifted off the slide and placed in lysis buffer, using a sterile needle, as necessary. Finally, if the tumor cellularity was very poor, laser capture microdissection of individual tumor cells was performed.

DNA was extracted using the High Pure FFPET DNA Isolation Kit (Roche) and quantified using the Invitrogen Qubit Fluorometer before genome-wide copy number analysis using WGS or SNP array (details below).

1. Fresh-Frozen Samples

Individual tumor glands were lysed in buffer ATL supplemented with proteinase K (Qiagen) during overnight incubation at 56°C. Genomic DNA was extracted using QiaAmp DNA Micro Kit (Qiagen) following the manufacturer’s protocol and further concentrated using Agencourt Ampure XP Beads (Beckman Coulter Inc.). Subsequently, DNA concentrations were fluorometrically determined using Qubit dsDNA HS Assay Kit (Thermo Fisher Scientific). Additionally, peripheral blood buffy coat DNA was prepared using the same strategy and used for germline comparison in downstream genomic analyses.

During the study, we became aware that isolated tissue we believed to be tumor glands during the microdissection often had very low or no tumor content when measured by genome sequencing and so may have been normal colon crypts or other glandular shaped tissue (perhaps stromal in origin) from the tumor. To preselect tumor glands for subsequent next-generation sequencing, TaqMan SNP Genotyping (Applied Biosystems) assays were designed for the specific *APC* or *BRAF* mutations previously identified by bulk exome sequencing of the tumor (see below for details). The TaqMan assays quantified the allelic fraction of somatic mutations in the *APC* or *BRAF* gene and thus served as a sensitive, quantitative measure of tumor cell content. All putative tumor gland DNAs from a single patient were collectively subjected to qPCR using the QuantStudio 7 system (Applied Biosystems), and matched DNA from bulk tumor tissue was used as a positive control. Only glands with detectable mutant allele signal were taken forward to next-generation sequencing library preparation and sequencing.

### Library Preparation and DNA Sequencing

1. Sequencing of DNA Derived from Archival Material

Prior to library preparation, the input DNA was subjected to repair using the NEBNext FFPE DNA Repair Mix (New England Biolabs). Library preparation for all samples was done using the NEBNext Ultra II FS DNA Library Prep Kit for Illumina (New England Biolabs). A fragmentation time of 5 minutes was used for the samples. The size selection protocol using Agencourt AMPure XP beads (Beckman Coulter) was applied only for samples with DNA input ≥100 ng going into the FFPE DNA repair step.

All libraries were indexed with unique indexing primers. Two sets of indices were used for the samples: NEBNext Multiplex Oligos for Illumina Dual Index Primers Set I and NEBNext Multiplex Oligos for Illumina 96 Unique Dual Index Primer Pairs (New England Biolabs). PCR enrichment of adapter-ligated DNA was done using the above-mentioned indices and the NEBNext Ultra II Q5 Master Mix (New England Biolabs). The standard protocol, as recommended by the manufacturer, was used for the entire library preparation with one variation across all samples—half volumes were used throughout the process.

The DNA libraries generated were first quantified using Qubit dsDNA HS (high-sensitivity) Assay Kit and Qubit 4 Fluorometer (Invitrogen) before running it on the Agilent TapeStation system using an Agilent High Sensitivity D5000 ScreenTape (Agilent Technologies). Subsequently, equimolar pooling and sequencing was done in preparation for sWGS. The pooled libraries were then subjected to sequencing (target genomic coverage 0.1x) either on a NextSeq 550 system or a NovaSeq 6000 system (Illumina).

1. Sequencing of Individual Tumor Glands

For the construction of sWGS libraries, DNA obtained from tumor glands was processed using NEBNext Ultra II DNA Library Prep Kit (New England Biolabs) according to the manufacturer’s instructions.

Whole-exome sequencing (WES) libraries were prepared from bulk tissue pieces or individual glands with the Agilent SureSelect XT low-input reagents in combination with the SureSelect Human All Exon V6 baits (Agilent) and unique dual indexing strategy. WES libraries were prepared from bulk tissue pieces using the Agilent SureSelect whole-exome reagents with the SureSelect Human All Exon V5 baits by Source Biosciences or by Nextera rapid capture whole-exome preparation, again according to the manufacturer’s instructions.

The concentrations of all libraries were quantified using Qubit dsDNA HS Assay Kit and Qubit 4 Fluorometer (Invitrogen). Library fragment size profiling was performed using the Agilent TapeStation system using Agilent High Sensitivity D1000 ScreenTapes (Agilent Technologies) and subsequently all libraries were pooled equimolarly.

sWGS libraries were subjected to shallow sequencing on a Illumina NextSeq 550 system using mid- or high-output kits (target genomic coverage: 0.1×), whereas WES libraries were subjected to deep sequencing on a NovaSeq 6000 system using a S2 PE100 flow cell (target exonic coverage: 300×) or a HiSeq 2500 platform (target exonic coverage 50×).

### SNP Arrays on DNA Derived from Archival Tissue

The quality of FFPE DNA extractions was verified using the Infinium HD FFPE QC Kit (Illumina), and then 100 to 250 ng of each sample was treated using the Infinium HD FFPE Restore Kit (Illumina) and processed using the Infinium HTS Assay, Manual Protocol (Illumina). Samples were then run on the HumanOmniExpress-24 version 1.1 BeadChip using the iScan system at the QMUL Genome Centre.

### FISH

Sections of FFPE tissue at 5-micron thickness were stained as previously described (41) using SureFISH probes against Chr8 CEP (Agilent, cat. no. G101034R-8) or Chr18 CEP (Agilent, cat no G101074R-8).

### Organoid Culture and Passaging

The 3994-117 patient-derived organoid (PDO) was established from a liver metastasis of a colorectal tumor, and it has been previously characterized as described in (30). PDOs were cultured embedded in Growth Factor Reduced Basement Membrane Matrix (Corning) and Advanced DMEM/F12 media (Thermo Fisher Scientific) supplemented with 1× B27 and 1× N2 supplements (Thermo Fisher Scientific), 0.01% BSA (Roche), 2 mmol/L L-Glutamine (Thermo Fisher Scientific), and 100 units/ml penicillin and streptomycin (Thermo Fisher Scientific). Additionally, 12 different growth factors were used to maintain PDO culture: 50 ng/mL EGF, 100 ng/mL noggin, 500 ng/mL R-spondin 1, 10 ng/mL FGF-basic, 10 ng/mL FGF-10 (all from PeproTech), 10 nmol/L gastrin, 10 μmol/L Y-27632, 4 mmol/L nicotinamide, 5 μmol/L SB202190 (all from Sigma-Aldrich), 100 ng/mL Wnt-3A (R&D Systems), 1 μmol/L prostaglandin E2, and 0.5 μmol/L A83-01 (Tocris Bioscience).

Passaging of PDOs was performed using TrypLE 1X diluted in 1 mmol/L PBS-EDTA (Thermo Fisher Scientific). In short, after media removal, PDOs in Matrigel were harvested by pipetting with 1 mL of TryplE1X, and they were incubated for 20 minutes at 37°C, with mechanical homogenization every 5 minutes. Then, PDOs were centrifuged at 1,200 rpm for 5 minutes at 4°C and washed with Hank’s Balanced Salt Solution (Thermo Fisher Scientific). Counting and viability measurements were done using 0.4% trypan blue staining solution (Thermo Fisher Scientific) and the Countess 3 Automated Cell Counter (Thermo Fisher Scientific). Expected cells were pelleted again and reseeded in Matrigel.

### Monolayer Cell Culture

The colorectal cancer cell line SW620 was obtained from the ATCC (CCL-227) and cultured in high-glucose DMEM with 10% FBS and 2% penicillin–streptomycin. Cells were grown in T-175 vented flasks at 37°C, 5% CO_2,_ and 95% relative humidity. Cell line identities were confirmed using short tandem repeat analysis at the beginning of the experiment and were tested regularly for *Mycoplasma* infection.

### Single-Cell WGS Using Direct Library Preparation + Method

For interrogation of SW620 single-cell whole genomes using the DLP+ method, the recently published protocol by Laks and colleagues (42) was adapted and optimized for a 384-well “one-pot” plate-based workflow.

First, 1 μL each of 384 unique i5 indexing primer at a concentration of 4 μmol/L were dispensed into empty 384-well plates and air-dried. Oe μL lysis buffer, consisting of 86.2% DirectPCR Lysis Reagent (Cell; Viagen Biotech, cat# 301-C), 8.6% protease (Qiagen, cat# 19157), and 5.2% glycerol (Sigma-Aldrich, cat# G5516-1L) was added to each well, and a single SW620 cell was sorted into each well using the CellenOne F1.4 system (Cellenion). Following centrifugation of the plate at 3,000 *g* for 5 minutes and overnight incubation at +4°C, cell lysis was performed by incubating the plate at 50°C for 1 hour and 70°C for 15 minutes. Tagmentation of genomic DNA was carried out by adding 1.8 μL tagmentation mix, consisting of 1.775 μL tagmentation buffer (20 mmol/L Tris-HCl, pH 8, 10 mmol/L MgCl_2_, and 20% dimethylformamide), 0.0165 μL Tween, 0.00875 μL Tn5 loaded (Diagenode, cat# C01070012-30), and incubating at 55°C for 10 minutes. Subsequently, the reactions were neutralized by adding 1 μL neutralization mix (0.5 μL Qiagen protease, 0.01 μL Tween, and 0.049 μL ddH_2_O) and incubating the plate at 50°C for 15 minutes and 70°C for 10 minutes. Finally, PCR amplification was performed after adding 3.8 μL master mix [3.78 μL 2× NEB Ultra II Q5 Master Mix (New England Biolabs, cat# M0544L) and 1 mmol/L i7 indexing primer (500 nmol/L final, Integrated DNA Technologies)] with the following cycling parameters: gap-filling at 72°C for 5 minutes, initial denaturation at 98°C for 30 seconds followed by 10 cycles of 98°C for 30 seconds and 65°C for 75 seconds, and final elongation at 65°C for 5 minutes. PCR products were pooled and purified using a Zymo DCC5 spin column and then subjected to exonuclease I digestion (New England Biolabs, cat# M0568) and 1× Ampure bead purification. Final libraries were quantified using Agilent HS D1000 screentapes on an Agilent TapeStation 4200 system and sequenced on the Illumina NovaSeq 6000 system using NovaSeq S2 PE50 flow cells.

### Single-Cell WGS Using 10X Chromium Single-Cell CNV

Prior to starting the 10× workflow, the cell viability was confirmed to be >90% using the Countess II Automated Cell (Thermo Fisher Scientific), targeting an estimated number of captured cells ranging between 1,500 and 3,000. The single-cell suspension was loaded on a Chromium Single Cell 3′ Chip C, followed by a Chip D (10× Genomics). Single-cell gel bead-in-emulsion was generated using the Chromium Single Cell DNA Kit and the Chromium Controller.

A scDNA-seq library was prepared, and the final library quality was confirmed using the TapeStation High Sensitivity ScreenTape assay D1000 (Agilent) and a High Sensitivity Qubit dsDNA Kit (Life Technologies). Samples were normalized, pooled, and sequenced in an Illumina NovaSeq 6000 according to standard 10× Genomics recommendations at a median depth of at least 750K read pairs per cell. The sequencing depth enables more than 2 Mb CNV detection per cell.

### Bioinformatic Processing

#### SNP Array Processing

The raw intensity values were normalized prior to calculating the logR ratios from the HumanOmniExpress-24 version 1.1 BeadChip. This standard normalization adjusts for systematic biases, including GC content, to ensure accurate comparisons between samples. For a subset of the samples matched normals were used to create a panel of normals (PON). Following this, the PON was used to calculate the log-R ratio for each sample. For 500 Kb bins across the genome, the mean log-R ratio was calculated and assigned to a single position within the bin to decrease noise within the sample and enable a direct comparison between the sWGS data (see below). The *winsorize* and *pcf* functions from the copynumber package (43) were then used to remove outliers and segment the data, respectively. For each sample, a density plot of the log-R ratios for the segments produced a number of normally distributed peaks, the greatest of which was assumed diploid normal. All values were diploid centered to this point. For those samples which were potentially triploid or tetraploid, the samples were diploid centered to the “correct” diploid peak for all multiregion samples from the same patient. These new log-R ratio values were then analyzed using the CGHcall package (SCR_001578), which adopts the DNAcopy (SCR_012560) method for segmentation. Each sample was preprocessed, normalized, segmented, and normalized after segmentation, and gains and losses were called.

#### Sequencing Quality Control

Per base quality, GC content, and adapter contamination of the raw read sequences in each fastq file were assessed using FastQC (versions 0.11.5 and 0.11.8; SCR_014583). Read clipping in a subset of samples that were found to have adapter contamination was performed using the Skewer tool (versions 0.2.1 and 0.2.2; SCR_001151). A minimum read length of 35 bp after trimming was required. Reads were aligned with Burrows–Wheeler Aligner (versions 0.7.15 and 0.7.5 using default parameters; SCR_010910) to UCSC human genome reference version 19. Genome build GRCh38 was used for the time course metastasis dataset in Fig. 4.

To check the quality of the alignments, a combination of Picard (SCR_006525) tools (*CollectWgsMetrics*, *CollectInsertSizeMetrics*, and *CollectAlignmentSummaryMetrics*, all versions 2.18 or 2.18.11) was used. Duplicate reads were marked using Picard *MarkDuplicates*. Of note, we ensured that the percentage of reads removed was not excessive (<35%) and that the target coverage and depth were obtained. Finally, on WES data, bamutil *ClipOverlap* version 1.0.14 was run to clip overlapping reads where insert sizes were small.

#### CNA Calling in sWGS Data

On sWGS, the R package QDNAseq (SCR_003174) was used to call CNAs. The QDNAseq package bins reads from the input BAM file into a user-specified series of bins, applies filtering (loess residual and blacklisting), corrects the bins for GC content and mappability, and then normalizes read counts per bin using the “median” method and smooths outlier counts.

QDNAseq was also used to segment per-bin read counts into “segments” of equal copy number. The “sqrt” function was used to transform the data, bin counts normalized with the “normalizeSegmentedBins” function.

To facilitate integration of SNP array data samples with the sWGS, 500 Kb bins were used in both platforms, and losses and gains for both data types were called using the CGHcall R package *calls* function. For data included in Fig. 1 in which sWGS and SNP array data were compared, 1 Mb bins were constructed for both sWGS and SNP array data, and the data segmented. Segments ≤2 Mb in length were deemed noise and removed.

For data in Fig. 4, 500 kb bins were used with no segment-size filtering. In Fig. 4, the resulting log_2_ ratio values in each bin as well as the segment mean of each bin were normalized according to the global median log_2_ ratio by subtracting this value from all log_2_ ratios.

#### Analysis of Exome Sequencing Data: Calling SNVs and Insertions and Deletions

A two-pass approach was used to identify SNVs and insertions and deletions (indels). First, to obtain a set of candidate variant “proposals” for each gland and bulk sample, each sample was run through GATK Mutect2 (GATK version 4.0.1; ref. 44). The nondefault parameter “–dont-use-soft-clipped-bases” was added to ensure the bases removed from Skewer and *ClipOverlap* were not reintroduced into the analysis. All resultant vcfs from each set were then merged into a single file representing the union of all variant proposals. A second variant caller, Platypus (version 0.8; ref. 45), was then used to assess the presence of each variant proposal in each gland and bulk sample. Platypus was designed to call haplotypes within genomes, making it suitable for use on the near-clonal glands used in this study. Variants marked with the “allele bias” filter flag were not excluded. The quality control (QC) parameters were set as follows: minMapQual = 20, minReads = 3, maxVariants = 100, and trimOverlapping = 0. To ensure equal calling across the samples, the following criteria for each variant was used: minimum depth = 10× and minimum variant allele frequency 5%. Note, if a sample failed QC (below), it was excluded from this filtering step.

#### Analysis of Exome Sequencing Data: Assessing SNV Pathogenicity

The platypus SNVs and indels (both somatic and germline) were annotated using AnnoVar (v 20190409; SCR_012821), SIFT (version 6.2.1; SCR_012813), and POLYPHEN (version 2; SCR_012776), the scores from which were obtained via VEP 92.5. Indels were assessed with VEST (within CRAVAT version 4.3; SCR_012776). This enabled putative driver mutations to be identified using the following criteria. If a variant was marked as pathogenic with *P* ≤ 0.05 by one of the above tools, or was a “STOPGAIN” and in a known hotspot [identified through the International Cancer Genome Consortium (ICGC) database], and was in a tier 1 COSMIC driver epithelial cancer census gene (see: https://cancer.sanger.ac.uk/cosmic/census?genome=37), then it was considered a potential driver.

#### Analysis of Exome Sequencing Data: Calling CNAs

The Sequenza R package (version 3.0; ref. 46) was used to call allele-specific CNAs. Initially a first-pass analysis on all samples was performed in which a broad range of potential cellularity (0.05–1) and ploidy (0.8–5) values were considered. Following manual inspection of the model fits and breakpoints of each sample, Sequenza was rerun with narrower priors covering the likely true cellularity and ploidy values. The second run also made use of a table of candidate breakpoints compiled from the aggregate across samples from that cancer of the first run. To construct the candidate table, we used a breakpoint merging routine (custom script) that merged first-round breakpoints that differed by less than a megabase in location.

#### Calling Absolute Copy Number where Paired Exome Sequencing Data Existed

Each colorectal cancer presented in Fig. 2 had both sWGS and exome-sequenced glands available. Absolute copy number information derived from Sequenza was used to infer absolute copy number of the sWGS data. Near-clonal CNAs (present in essentially every gland) were assumed to have the same copy number as inferred in the exome sequenced gland(s). The copy number of other segments was then inferred using these known copy numbers as a reference point.

#### Calling Absolute Copy Number from sWGS Data in the Absence of Exome Sequencing Data

In Fig. 4, we followed the approach laid out by the Allele-Specific Copy Number Analysis of Tumours (ASCAT) (47); however, as we only had log_2_ ratio values available due to the low coverage of the data, we sought to leverage multiple sampling to search for ploidy solutions. Here, we search across a range of ploidies (1.5–4), allowing solutions to be found with a minimum purity of 0.5 for laser microdissected “region” samples and 0.2 for “bulk” samples. We recorded the per-sample fit for each ploidy value. We then recorded the pairwise Euclidean distances of the integer copy number values of each sample divided by the ploidy, filtering for bins with a log_2_ ratio greater than 1 to avoid distances being exaggerated by amplifications. To determine the ploidy of the patient, we compared the mean pairwise Euclidean distances of ploidy normalized copy number values to the mean per sample solution for each ploidy value after linearly scaling each measure to the maximum and minimum values, (i.e., minimum equals 0 and maximum equal 1). Ploidy solutions were then ranked according to their Euclidean distance from both values being zero. This was performed to ensure the chosen ploidy value produces a good fit both within samples and across samples.

After manual curation, the second-best fitting option was chosen for LM0032 and LM0082. Additionally, Liv-C-R1 in LM0131 was determined to be less than 50% pure and was refitted accordingly. The ploidies for fitting copy number profiles of samples Rec-B-B2 (LM0001) and Liv-C-R2 (LM0131) were increased by 0.4 and Liv-G-R1-3 (LM0131) and Liv-B-R1 (LM0089) were decreased by 0.4 to accommodate outlier ploidies in these samples that produced poor fits.

### Sample QC

To ensure only samples with adequate tumor content and quality were included in downstream analysis, a sample QC step was introduced.

For exome sequencing, this was: (a) Sequenza-inferred tumor content (see above) minimum 70%, (b) SNV counts >70, and (c) clonal mutations with variant allele frequency > 0.2.

For the SNP array analysis, the variance of the logR values of bins within putatively diploid segments was considered. Samples that had variance that fell in the top 5% of the population of samples were removed. In addition, samples were removed when the logR values of bins in putatively diploid segments were not normally distributed around the mean (Shapiro–Wilks test). A further six samples were removed because of low cellularity.

### Phasing of WES and sWGS Copy Numbers

An approach similar to published methods (11, 48) was used to phase SNVs. In the cases presented in Fig. 2, information from WES and sWGS was available from the same tumor. Information was combined from these two data types to phase alleles. First, the set of SNPs that were detected in all exome sequenced glands was identified. These SNPs were subsetted to leave only those in chromosome copy-change regions, and the distribution of the B-allele frequencies (BAF) was plotted. SNPs phased to either the major or minor chromosomes (i.e., maternal and paternal chromosome) could be observed by being at high vs low BAF, respectively. For instance, in a trisomy region the allele imbalances resulted in SNPs shifted to occupy the 0.33 (minor) and 0.67 (major) ranges when purity was 1.

Where coverage permitted, these phased SNPs were then recaptured in each of the sWGS samples. The total number of bases containing either the A or B allele of the SNPs was then used in a statistical analysis that aims to build evidence for the same BAF shift as observed in the exome gland exomes. We used binomial statistics to test whether a significant departure from 0.5 frequency exists in the ratio of the A and B alleles. Note that in addition to verifying the copy calls produced from the QDNAseq method, this phasing routine effective allows us to call copy neutral loss of heterozygosity events in sWGS data as well.

Phased SNPs were then considered in the sWGS data. For each CNA segment, the number of detected major-allele and minor-allele SNPs was reported, and we note that most SNPs were undetected due to low coverage. To test for the expected allelic imbalance, the segmental SNP counts were then assessed with a Fisher exact test with the null expectation based upon the inferred allele-specific copy number.

### Phylogenetic Tree Construction from SNVs

PAUP* software (SCR_014931) was used to build phylogenetic trees from SNV data (Fig. 2). Variants were represented as a binary matrix, in which the rows corresponded to a particular sample or the normal sample and the columns to a specific variant and binary encoding (0/1) indicated absence or presence of a variant.

A nexus file was constructed for each set to specify the parsimony search parameters as follows: (i) the outgroup function was used to “root” the phylogenies in respect to the normal sample; (ii) the *hsearch* function was used to heuristically search 10,000,000 trees; we retained 1,000 of the shortest trees; (iii) the bootstrap routine was used for subsampling the tree construction and data 10,000 times. This involved randomly selecting a set of mutations from the binary matrix (with replacement). The percentage of each branch instance was reported in a log file and used to annotate the trees; and (iv) the *alltrees* function was used in sample sets with less than 10 samples. This “brute-force” run makes it possible to acquire the definitely shortest tree(s) from the total tree-space. The resulting .tre files were inspected and converted to .pdf format using FigTree (SCR_008515). The homoplasy indexes for the trees were calculated and output as part of the PAUP* log file.

To obtain a *P* value for tree balance, the Colless test function in the R apTreeShape package (49) was used. The statistics represent comparison against a “Yule” growth process, meaning the *P* value represents the likelihood of imbalance compared with the null expectation provided by this model.

### Assessment of CNA Divergence

Divergence of CNAs across samples within each patient was quantified by computing the pairwise divergence between samples. Specifically, this was the proportion of altered bins (copy number not equal to the base line ploidy in either or both samples) that had different copy numbers in each sample. When multiple samples were available from each patient, the distribution of all pairwise comparisons was computed, and statistical tests on subsets of comparisons were performed as described in the main text. This measure of divergence was comparable with others. The following measures were compared for similarity and presented in Supplementary Fig. S2:

1. **Fraction of different bins** (FracDiffBins), defined by counting the number of bins with different copy number status in the two samples (gained, lost, and baseline) and dividing by the total number of bins across the genome.
2. **Fraction of different altered bins** (FracDiffAltBins), defined by counting the number of bins with different copy number status (gained, lost, and baseline) and dividing by the total number of bins that are not baseline in at least one of the two samples in a pair. This is presented in the main article, where it is called “proportion of aberrant genome subclonal.”
3. **Fraction of different bins weighted by gene content** (GeneDoseFDB) is the same as (1); however, each bin is weighted by the number of genes it contains (including the total number of bins) to explore whether gene-rich/poor areas are increasing/decreasing diversity measurements.
4. **Fraction of different altered bin weighted by gene content** (GeneDoseFDAB) weights each bin by its relative content of genes (weight = number of genes in the bin) and uses the weights to compute diversity as per metric (3).
5. **Genetic distance** is the sum of the difference between integer copy numbers in each bin as opposed to a binary same/different criteria for baseline, gains, and losses. The total sum is divided by the number of bins across the genome.
6. **Breakpoint divergence** measures the amount of nonoverlapping breakpoints as a proportion of the total breakpoints present in a pair of samples.

All diversity statistics were highly correlated with one another (Supplementary Fig. S2). Importantly, the correlation between statistics that normalized for the proportion of the altered genome and those that did not (e.g., FracDiffBins v FracDiffAltBins) was very high (Pearson correlation coefficient = 0.742), showing that our measurement of low CNA diversity in cancers relative to adenomas was not an artefact of the choice of statistic.

Linear mixed-effects models that control for interpatient differences were calculated using the lmerTest package (SCR_001905) in R.

#### Genome Duplication Classifier

Using ICGC segment calls, we binned the genome into 4 Mb bins to mimic data expected in low-coverage WGS. In this study, we took the median calls for each bin when comparing overlapping segments. For each sample, we calculated the number of segments after binning by detecting changes in call status in adjacent bins across the genome. We calculated PGA by measuring the fraction of bins across the genome that did not equal the median copy number of the bins of the sample (baseline copy number). Taking the called GD status per sample from the ICGC resource we trained a support vector machine using the e1071 package (10.32614/CRAN.package.e1071) in R (no scaling, a linear kernel, and a cost of constraints violation of 5). The classifier to these data calls GD as present in a sample if

0<0.000355NS+PGA-0.432 (1)

[In which NS denotes the number of segments and PGA denotes the percentage genome altered (fraction of genome with nonbaseline copy number)]. On PCAWG data, this classifier calls 83.8% of GD samples correctly, and 93.2% of non-GD correctly (Supplementary Fig. S4).

We determined an “ambiguous” call zone ±0.5 of the beta0. To classify patients with multiple samples patients were considered clonally non-GD if at least one sample was “confidently” called non-GD and no samples were “confidently” called GD and vice versa. Subclonal GD was called when a patient has both “confident” non-GD and GD calls. Ambiguous calls per sample were then corrected according to the patient assessment, in subclonal GD patients, ambiguous calls were corrected to the confident call that had the highest frequency in the patient, and if this was tied, we corrected to ambiguous calls to GD.

#### Phylogenetic Analysis of sWGS Data

Copy number data across all samples for a given patient were split up based on breakpoints detected in at least one sample (defined as a copy number difference between two consecutive bins) to generate a comparable set of regions. Regions containing less than 4 bins were removed for phylogenetic analysis. Phylogenetic trees were created using MEDICC2 (34) in the total copy number model. Trees were plotted using ggtree (50). Reconstructed MEDICC2 unobserved copy number states were used to calculate clonal and tip frequencies. Clonal changes represented gains and losses from the root, and tip changes presented alterations present between the tip nodes and the nearest internal node. Gains and losses were only counted once even if they resulted in a change in copy number greater than 1 and therefore more than 1 copy number event. Clonal events were normalized to the ploidy of the patient to avoid all regions being gained in triploid and tetraploid tumors.

#### Copy Number Quantification in DLP+ scDNA-seq

Demultiplexed dual-index fastq files were obtained for single cells undergoing the DLP+ protocol, and the data were used as input for the workflow automation pipeline designed for the DLP+ method (https://github.com/shahcompbio/single_cell_pipeline) using default settings. First, raw reads were adapter-trimmed using TrimGalore, mapped with Burrows–Wheeler Aligner-aln to the hg19 reference genome, and deduplicated using Picard MarkDuplicates. Subsequently, copy number calling was performed using the HMMcopy tool with reads segmented into nonoverlapping 500 kb genomic regions. Cells were required to have a minimum quality score of 95, a minimum of 250,000 reads, and an S-phase probability of less than 0.1. Only bins considered “ideal” in all cells were used.

#### Copy Number Quantification in 10X scDNA-seq

As per the 10X recommended analysis, CNAs were determined using CellRanger with default settings (using GRCh38 as the reference genome). For each cell, the mean event confidence weighted by segment size and number of segments was calculated, a minimum mean event confidence of 10^1.85^ was required per cell, and a minimum of 416 segments was allowed. Calls were then binned into 1 Mbp bins, and bins with no overlapping segments in at least one cell were removed. Cells with more than 50% of the genome with copy number zero were removed, as were cells with a mean copy number of more than 20. A maximum of 105 segments based on final bins were allowed per cell.

#### Phylogenetic Analysis of scDNA-seq Data

Comparable regions were calculated for the SW620 and 3994-117 cells according to the sWGS approach. For both sets of cells, ploidy was limited to less than the mean ploidy + 1 to remove potential G2 phase cells or doublets. A minimum of 2 bins was required for regions for both populations. A random sample of 100 3994-117 cells were selected to construct the tree to make it comparable with the SW620 tree. MEDICC2 was used, trees were plotted, and clonal and tip frequencies were calculated, as previously described for sWGS.

### General Statistical Analyses

All statistical comparisons were performed in the R statistical programming language (SCR_001905), with specific statistical tests used described in the article.

### Mathematical Modeling of CNA Evolutionary Dynamics and Statistical Inference

To model the fitness effects of chromosomal instability, a computational framework called *CINulator* was developed. *CINulator* models tumor evolution as a birth–death process in which cells can gain or lose loci at a specified rate μ. Changes in copy number at a locus may influence the birth and/or death rates. Fitness is incorporated by defining a fitness landscape in which the optimum is some prespecified genotype $G_{O}$, the fitness of individual cells is proportional to the distance from this optimum genotype:

$$s=\frac{s_{\max}}{1+\alpha \left|G_{O}-G_{i}\right|}$$

Fitness defined as the net growth rate s=b-d. Changes in fitness may either increase the birth rate or decrease the death rate. The simulation begins with a single cell that has some genotype G which can be at, or close to, the optimum. In this scenario, the majority of changes will bring a fitness cost. Alternatively, if the genotype of the founder cell is far away from the optimum, then the majority of changes will give a fitness benefit, and the population will tend to climb the fitness landscape.

We also constructed a Bayesian inference framework, based on an Approximate Bayesian Computation method, to compare model predictions with data and learn the model parameters that best described the observed data.

A full description of the model and inference framework is provided in the Supplementary Modeling Note, and details of the various assessments of model dynamics and statistical approach are provided.

## Supplementary Material

Supplementary Note on Modelling 1 Supplementary note where we describe the gland mathematical modelling and simulations

Supplementary Table 1 Pairwise comparison metrics for data presented in Figure 1.

Supplementary Table 2 Per genomic bin copy number calls (gains=3, diploid=2, loss=1) for data presented in Figure 1.

Supplementary Table 3 Clinical information for CRC cohort presented in Figure 1.

Supplementary Table 4 Per patient clinical information for the CRC cases presented in Figure 2.

Supplementary Table 5 Per segment raw sWGS copy number data for patients presented in Figure 2.

Supplementary Table 6 Per segment Sequenza copy number data and calls for patients presented in Figure 2.

Supplementary Table 7 Annotated somatic mutation calls derived from exome sequencing data across multiple regions in patients presented in Figure 2.

Supplementary Table 8 Per genomic bin copy number calls for single-cell data presented in Figure 3.

Supplementary Table 9 Cohort information per sample for the longitudinal metastasis cohort (Figure 4), including description of organ, timepoint sampled, tumour stage at diagnosis, whether the sample is treatment naïve, if the sample was treated in the prior interval, the number of days and months elapsing since the first timepoint, in addition to per sample genomic metrics such as PGA, number of segments, patient ploidy, per sample GD classification and overall patient GD classification.

Supplementary Table 10 Per sample, per segment, raw copy number data (log2 ratios) and integer calls for the longitudinal metastasis cohort presented in Figure 4.

Supplementary Figures S1-S17 Supplementary Figures 1-17 and Figure Legends Supplementary Figure 1: CNA subclonality decreases with progression within early colorectal cancers. Supplementary Figure 2: CNA subclonality assessed by different metrics. Supplementary Figure 3: Divergence decreases with progression within early colorectal. Supplementary Figure 4: Derivation of a simple genome doubling classifier. Supplementary Figure 5: Divergence is highest in subclonal and lowest in clonal genome doubled cases. Supplementary Figure 6: In situ analysis using FISH and DNAscope shows copy number instability at the single-cell level. Supplementary Figure 7: Simulations demonstrate stabilising selection suppresses karyotypic diversity when tumours are close to fitness peak. Supplementary Figure 8: The non-identifiability of the mutation rate (μ) and the strength of stabilising selection. Supplementary Figure 9: Cross-cohort study shows CNA subclonality, CNA burden and genome doubling change with progression. Supplementary Figure 10: Multi-site longitudinal karyotype phylogenetic assessment of individual CRC patients during tumour progression and drug treatment. Supplementary Figure 11: Multi-site longitudinal karyotype assessment of individual CRC patients during tumour progression and drug treatment (patients LM001 – LM0029). Supplementary Figure 12: Multi-site longitudinal karyotype assessment of individual CRC patients during tumour progression and drug treatment (patients LM0032 – LM0089). Supplementary Figure 13: Multi-site longitudinal karyotype assessment of individual CRC patients during tumour progression and drug treatment (patients LM0095 – LM0139). Supplementary Figure 14: Number of phylogenetic copy number events and proportion of the aberrant genome subclonal correlate in carcinomas. Supplementary Figure 15: Patterns of aneuploidy evolution during metastatic spread and treatment. Supplementary Figure 16: Recurrent CNAs are similar between the primary and metastatic lesions. Supplementary Figure 17: Early colorectal cancer and primary tumour samples from metastatic patients show similar CNA recurrence and number of segments, but not PGA.

## Data Availability

Processed data sufficient to reproduce the analysis in each figure are presented as supplementary tables, referenced throughout the article. All raw sequencing data and arrays files will be available through the European Genome–phenome Archive repository, accession number: EGAS00001004219. Code used for analysis is available on GitHub: https://github.com/BCI-EvoCa/CNA_stability, and simulation code is available at https://github.com/ucl-cssb/CIN_CRC.
